# A Microfluidic Framework for Neuroprotective Compound Triage Across Ischemia and Neurodegeneration

**DOI:** 10.3390/molecules31101622

**Published:** 2026-05-12

**Authors:** Julia Anchimowicz, Slawomir Jakiela

**Affiliations:** Department of Physics and Biophysics, Institute of Biology, Warsaw University of Life Sciences, 02-787 Warsaw, Poland; julia_anchimowicz@sggw.edu.pl

**Keywords:** microfluidics, organ-on-a-chip, nervous-system organ-on-a-chip, blood-brain barrier, neurovascular unit, ischemia-reperfusion, stroke, neurodegeneration, mitochondria, phenotypic screening

## Abstract

Microfluidic systems are increasingly used in neuroprotection research, but their clearest value may be to show why candidate compounds fail before costly downstream models. This critical framework review examines CNS-relevant microfluidic studies through a within-program triage logic linking chemistry-aware prescreening, blood-brain barrier/neurovascular unit (BBB/NVU) filtering, and timed validation in neuronal ischemia/reperfusion models, and treats non-CNS organ-on-a-chip and analytical microfluidic studies as engineering analogies only. The available evidence most strongly supports BBB/NVU chips as exposure- and safety-aware filters and compartmentalized neuronal oxygen-glucose deprivation platforms as timing-sensitive validation tools; droplet microfluidics contributes mainly upstream through dense dose mapping, aggregation assays and counterscreens for assay interference. A compound-centered reading also suggests that apparent activity often fails for distinct reasons, including timing mismatch, poor solubility, surface adsorption, optical artifact, inadequate multicellular context, or loss of efficacy under transport-aware testing. Taken together, the literature supports a cautious, within-program triage logic in which microfluidics is used not as a universal disease model, but as an operational framework for exposing transport, barrier, timing and assay liabilities early in neuroprotective discovery.

## 1. Introduction

Neuroprotective therapies still often lose effectiveness when they move beyond simplified preclinical assays. This is most visible in acute ischemic stroke, where many candidates look promising in static cultures or animal models but fail to deliver robust clinical benefit.

A related but distinct problem occurs in chronic neurodegenerative disorders. Disease progression is slower, causation is multifactorial, and neuronal injury depends on sustained vascular, glial and immune interactions. These features call for models that reveal delivery, timing, multicellular context and mechanism-linked failure before costly downstream testing.

Several recent reviews have summarized microfluidic approaches for neurological disease modeling and have provided valuable guidance on neural circuits, BBB/NVU chips and screening workflows [1,2,3,4,5,6,7,8,9,10,11]. However, the central question for neuroprotective discovery is not simply which platforms are available, but where they can change compound decisions earlier than conventional workflows.

Microfluidic and organ-on-a-chip systems are attractive in this context because they combine physical control and biological integration in ways that standard plate-based assays rarely achieve. By operating at the scale of capillaries, axon bundles and tissue microdomains, microfluidics enables stable laminar flow, defined shear, programmable gradients and spatial compartmentalization with low reagent use [12,13,14]. Organ-on-a-chip concepts extend these capabilities to coupled vascular, immune and neural modules that can model barrier transport, dynamic dosing and multicellular signaling while remaining compatible with imaging and electrophysiology [15,16]. For neuroprotective discovery, the importance of these systems lies less in a generic claim of increased physiological realism than in their ability to test whether exposure, timing and multicellular context materially alter the interpretation of a candidate compound.

This review, therefore, takes a selective, compound-centered perspective. Rather than cataloging microfluidic platforms, it asks where microfluidics improves triage by exposing failure modes earlier than standard workflows can. The primary evidence base is organized around three decision layers: chemistry-aware prescreening, BBB/NVU exposure filtering, and neuronal ischemia/reperfusion validation. Organoids, extracellular-vesicle workflows, active-field sample preparation, integrated sensing and proteinopathy-oriented assays are considered only where they sharpen one of those decisions. Thus, the Review is not a chemotype-by-chemotype pharmacology catalog, but a practical framework for identifying the earliest informative microfluidic test for different classes of bioactive compounds. The term compound-centered is used here in an operational sense: the platform is judged by whether it changes a go/no-go, re-formulation, re-ranking, or mechanistic follow-up decision for a candidate compound, not by whether it is the most complex disease model available.

For readability, the framework can be reduced to three practical questions. First, is the chemistry sufficiently stable and assayable to justify biological testing? Second, does the compound reach the relevant brain-facing or vascular compartment without unacceptable barrier liability? Third, does the apparent benefit persist when injury timing, delayed treatment and compartment-specific vulnerability are made explicit?

A central premise is that successful neuroprotection depends on aligning three domains often separated in conventional workflows: i) the delivery of substances across the BBB and related transporter constraints; ii) the timing and cellular context of injury, and iii) mechanism-linked readouts capable of distinguishing genuine rescue from assay-specific artifact. BBB dysfunction is relevant not only to drug exposure but also to injury biology itself [17,18,19]. Neuroinflammation and endothelial-pericyte-astrocyte signaling can amplify, mask or redirect compound effects depending on treatment timing and biological context [20,21]. Mitochondria are particularly informative because they integrate energetic failure, redox stress, Ca^2+^ dysregulation and proteostatic burden across multiple cell types [22,23,24]. However, they are most useful when interpreted together with barrier and structural endpoints rather than in isolation.

In this Review, neuroprotection is defined operationally as preservation of a specified injury-relevant endpoint (e.g., neuronal survival, axon/neurite integrity, mitochondrial recovery, barrier function, neuroimmune balance, or network activity) under explicit timing and exposure conditions. A positive result in one endpoint is therefore not treated as proof of global neuroprotection unless it remains coherent with orthogonal readouts and the intended compartment of action.

This logic also explains why ischemia and selected neurodegenerative contexts are considered together here, but only within clear limits. The overlap is most persuasive at the level of shared stress modules, including barrier dysfunction, inflammatory amplification, mitochondrial injury and impaired trafficking, rather than at the level of disease equivalence. Stroke, Alzheimer’s disease, Parkinson’s disease and broader proteinopathies remain distinct in timescale, cellular choreography and clinically meaningful endpoints. Those distinctions are therefore kept explicit throughout, even where microfluidic systems offer a useful common language for testing how compounds behave under transport-aware, time-resolved and multicellular conditions.

Viewed in this way, microfluidic platforms occupy a strategically useful middle ground between reductionist culture systems and expensive, low-throughput animal studies. Their clearest contribution is not miniaturization itself, but earlier separation of compound failure modes. Perfusion, shear, gradients and compartmentalization change how cells experience injury and drug exposure, making it possible to assess barrier transport, glial modulation, delayed dosing windows and axonal vulnerability under defined conditions [12,13,14]. This is especially important in neuroprotective discovery, where an apparently promising compound may fail because it never reaches the brain-facing compartment, is lost to materials, acts only as pretreatment, or loses efficacy once a more complete neurovascular context is restored.

Accordingly, this Review is structured around within-program triage logic rather than platform novelty. Its purpose is not to present microfluidics as a universal surrogate for neurological disease, but to identify where current evidence already supports compound-relevant decisions, where the strongest direct CNS support exists, and where the field still relies on plausible but only partially benchmarked engineering logic. The underlying argument is practical: the greatest value of microfluidics in neuroprotective discovery may be that it makes transport failure, barrier liability, timing mismatch, assay artifact and context-dependent loss of efficacy visible before compounds progress into more costly models.

## 2. Scope and Evidence Framework

This Review was designed as a critical framework review rather than as a PRISMA-style systematic review. Literature searches were conducted in PubMed/MEDLINE, Web of Science Core Collection, Scopus, IEEE Xplore and Google Scholar and were updated through March 2026. The search window extended from 2000 onward, but comparative interpretation was deliberately weighted toward contemporary CNS-relevant studies, with primary emphasis on microfluidic and organ-on-a-chip papers from 2022 to March 2026. Older studies were retained selectively when they established concepts that still define present assay logic, including BBB biology, oxygen-glucose deprivation paradigms, neuronal compartmentalization, droplet operations and transport measurement.

The review addresses one practical question: where microfluidic systems change compound decisions earlier than conventional workflows. Eligible literature therefore, included peer-reviewed original studies, benchmark papers, meta-analyses and reviews relevant to BBB/NVU chips, neuronal and neuron-glia microfluidic injury models, droplet-based prescreening, mitochondria-linked readouts and selected enabling technologies. However, direct CNS microfluidic primary studies were given the greatest weight in comparative claims. Disease-biology papers and translational compound reviews were used to frame mechanism and clinical caution, whereas non-CNS organ-on-a-chip, epithelial-transport and broader analytical microfluidics studies were included only when they clarified an engineering principle directly transferable to neuroprotective testing. These adjacent literatures were treated as engineering analogies, not as direct validation of neuroprotective workflows.

Evidence was synthesized narratively, with direct CNS microfluidic studies carrying the greatest interpretive weight. The analysis was structured around a staged decision sequence comprising chemistry-aware prescreening, upstream microfluidic prescreens, BBB/NVU exposure filtering, neuronal ischemia/reperfusion validation and mechanistic follow-up. For primary studies, attention was focused on factors most likely to alter compound interpretation, including device geometry and materials, flow and shear conditions, cell sourcing and multicellular composition, exposure fidelity, adsorption, oxygen/glucose control, timing of injury and treatment, and the use of orthogonal validation readouts. Because validated risk-of-bias tools remain limited for microfluidic platform studies, a formal PRISMA risk-of-bias score was not applied. Instead, we added a semi-quantitative evidence-confidence check to make the qualitative labels transparent. Direct CNS relevance, model validation/benchmarking, independent-group or inter-laboratory reproducibility, compound-decision relevance, and exposure/transport fidelity were considered as appraisal dimensions. Claims were treated as established only when several of these dimensions were met across direct CNS studies; emerging/moderate claims reflected thinner or heterogeneous direct evidence; and limited/framework-level statements indicated hypotheses assembled from plausible engineering logic but not yet prospectively benchmarked. Established claims required recurring support from multiple direct CNS studies across multiple models or groups; emerging claims reflected thinner or more heterogeneous direct evidence; and framework-level statements were reserved for workflow proposals assembled from partially mature evidence streams.

This approach should therefore be read as an evidence-guided synthesis, not an exhaustive ranking of all microfluidic neuroprotection studies. Its purpose is to identify where current evidence already supports compound-relevant decisions, where recurrent liabilities are visible across compound classes, and where the field still relies more on plausible engineering logic than on direct benchmarking. Accordingly, “decision value” denotes a reduction in a specific uncertainty that can change compound handling within a program--for example, advancement, de-prioritization, re-formulation, altered dosing window, or targeted mechanistic follow-up--rather than a claim that the platform has already improved clinical translation.

## 3. Shared Stress Modules Across Ischemia and Neurodegeneration: Where the Analogy Stops

### 3.1. Convergent Mechanisms and Disease-Specific Limits

The overlap between ischemia and chronic neurodegeneration is most useful at the level of shared stress modules, not as a claim that these disorders are biologically equivalent. BBB dysfunction, inflammatory amplification, mitochondrial injury, axonal transport stress and proteostatic burden recur across both acute and chronic settings, but they unfold on different timescales and within different cellular programs [15,16,17,18,25,26,27]. That distinction matters. A shared mechanism can justify a shared experimental question, but it does not automatically justify a shared assay logic.

This is where the ischemia-to-neurodegeneration analogy is most informative. In stroke, BBB opening, endothelial activation and neurovascular failure worsen edema, leukocyte recruitment and secondary injury [17,19,25]. In Alzheimer’s and Parkinson’s disease, vascular dysfunction is increasingly viewed as an early modifier of clearance, inflammation and network resilience [25,28,29]. It remains unjustified to treat these disorders as interchangeable simply because they intersect at a few stress nodes. Their clinically meaningful endpoints, treatment windows and multicellular choreography remain different, and model choice should stay disease-question-specific.

Mitochondria form a second point of convergence. In neurons, oxidative phosphorylation is essential for synaptic transmission and ion homeostasis; when it fails, ATP depletion accelerates excitotoxic injury and weakens recovery. During ischemia, collapse of Δψm, ROS generation and defective energetic recovery are central injury events [29,30]. In chronic neurodegeneration, altered fusion-fission balance, impaired mitophagy and disrupted axonal transport sustain vulnerability over longer intervals [31,32]. The overlap is therefore mechanistic, not nosological.

### 3.2. What “Neuroprotection” Means in Microfluidic Assays

Microfluidic neuroprotection assays should be explicit about what is being protected, in which compartment, and at what stage of injury. Protection may mean neuronal survival, preservation of neurite or axon integrity, maintenance of barrier function, dampening of inflammatory signaling, stabilization of mitochondrial physiology, or preservation of network activity [33,34]. These outcomes need not move together.

The timing of protection is equally important. A compound may look beneficial during OGD, lose efficacy when added at reperfusion, or improve one endpoint while worsening another during prolonged recovery. This is precisely where microfluidics becomes useful: it can impose defined transitions in oxygen, glucose, flow and dosing, and it can connect cell-intrinsic, tissue-level and system-level readouts within the same experimental logic [3,33,34,35].

One practical framing is staged resilience: (i) cell-intrinsic resilience, including mitochondrial and redox stability; (ii) tissue-level resilience, including neurite integrity and network function; and (iii) system-level resilience, including barrier performance and neuroimmune balance. This framing keeps “neuroprotection” from collapsing into a single convenience endpoint and supports the staged screening framework proposed later.

### 3.3. Compound Families, Negative Lessons, and Recurring Translational Liabilities

The compound landscape relevant to microfluidic neuroprotection is best organized by failure mode, not chemistry catalogs alone (see Table 1). Some candidates are intended to intercept acute oxidative or excitotoxic injury, others to stabilize the BBB or reshape neuroinflammation, and others to preserve mitochondrial quality control or proteostasis. These classes fail for different reasons. A review that remains platform-centered but ignores those differences risks mistaking elegant device design for pharmacological insight.

Acute radical scavengers provide the clearest historical lesson. NXY-059 remains the cautionary example: chemically plausible, preclinically encouraging and ultimately unsuccessful clinically once exposure, timing and patient heterogeneity became decisive [36]. Edaravone is a more complex counterexample. Meta-analytic syntheses support benefit in selected stroke settings, but they do not remove the class problem of narrow timing dependence and context sensitivity [37]. For microfluidic workflows, this family argues for early tests that combine realistic reperfusion windows with barrier-aware exposure rather than relying on pretreatment rescue alone.

Pleiotropic natural products such as resveratrol and curcumin remain attractive because they touch several linked pathways at once, including oxidative stress, inflammation, mitochondrial signaling and, in some cases, BBB stability [27,38,39,40]. They also illustrate why compound-centered microfluidics matters. Poor aqueous solubility, uncertain brain exposure, adsorption to device materials, autofluorescence, quenching, formulation dependence and hormetic dose-response behavior can all distort interpretation. A simple neuronal plate assay can therefore overstate promise unless free concentration, optical interference and transport are checked explicitly.

Two additional families reveal the opposite asymmetries. Barrier- or neuroimmune-modulating compounds may look weak in neuron-only assays yet become more compelling once endothelial injury, pericyte signaling and microglial activation are restored [41,42,43,44,45,46,47]. Mitochondria-directed agents, including peptides such as elamipretide and modulators of fusion-fission or mitophagy logic, raise a different question: do they still work when delivered after injury has begun and is improved survival accompanied by coherent recovery in Δψm, ATP, trafficking and quality control [32,48].

**Table 1:** Compound-led failure modes in neuroprotection and the earliest informative microfluidic decision test.

Compound Family/Representative Examples	Most Informative Early Question	Common False-Confidence Route in Conventional Assays	Earliest Informative Microfluidic Decision Test (Confidence Tier)
Acute radical scavengers/redox modulators (e.g., NXY-059, edaravone)	Does benefit persist at clinically plausible reperfusion timing and free brain-facing concentrations?	Pretreatment rescue; poorly defined exposure; benefit disappears once the dosing window narrows.	BBB/NVU transport under flow plus reperfusion-timed neuronal dosing. Evidence weight: moderate to strong for timing/exposure logic, but not for universal class success. Representative support and limits: clinical caution and timing/exposure logic [33,34,35,36,37,43,49,50].
Pleiotropic natural products and polyphenols (e.g., resveratrol, curcumin)	Is activity still present after correcting for solubility, adsorption and optical artifact?	Autofluorescence/quenching, adsorption to PDMS, formulation-dependent exposure, hormesis.	Droplet dose grids and counterscreens, then BBB/NVU triage before complex neuronal assays. Evidence weight: moderate. Representative support and limits: natural-product mechanisms plus adsorption/interference concerns [27,38,39,40,51,52,53,54,55,56,57].
Mitochondria-directed agents (e.g., elamipretide; fusion/fission or mitophagy modulators)	Do delayed dosing and multiparametric mitochondrial rescue remain aligned?	Apparent benefit only as pretreatment; survival endpoint improves without coherent mitochondrial recovery.	Dynamic neuronal OGD/reperfusion chips with multiparametric mitochondrial readouts. Evidence weight: moderate. Representative support and limits: mitochondrial mechanisms and neuronal-chip readouts [24,32,48,58,59,60,61,62].
Barrier- and neuroimmune-modulating compounds	Is benefit indirect (NVU preservation/inflammatory reprogramming) rather than neuron-intrinsic?	Neuron-only assays underrate compounds; modest direct neuronal potency is mistaken for lack of value.	Tri-culture BBB/NVU chips with TEER/Papp plus cytokine and microglial or leukocyte readouts. Evidence weight: emerging. Representative support and limits: NVU/neuroinflammatory context [41,42,43,44,45,46,47,49,63,64,65].
Anti-aggregation/proteostasis modulators	Does biochemical activity survive transport, trafficking and cellular clearance?	Cell-free hit does not translate to living neurons; chronic-timescale mismatch is ignored.	Droplet aggregation or condensate prescreens followed by neuronal-chip confirmation. Evidence weight: emerging to limited for direct neuroprotection translation. Representative support and limits: droplet aggregation and neuronal propagation assays [3,51,52,53,54,55,56,66,67].

## 4. Core Microfluidic Modules with Decision Value: What Remains Adjunct

**Figure 1:**
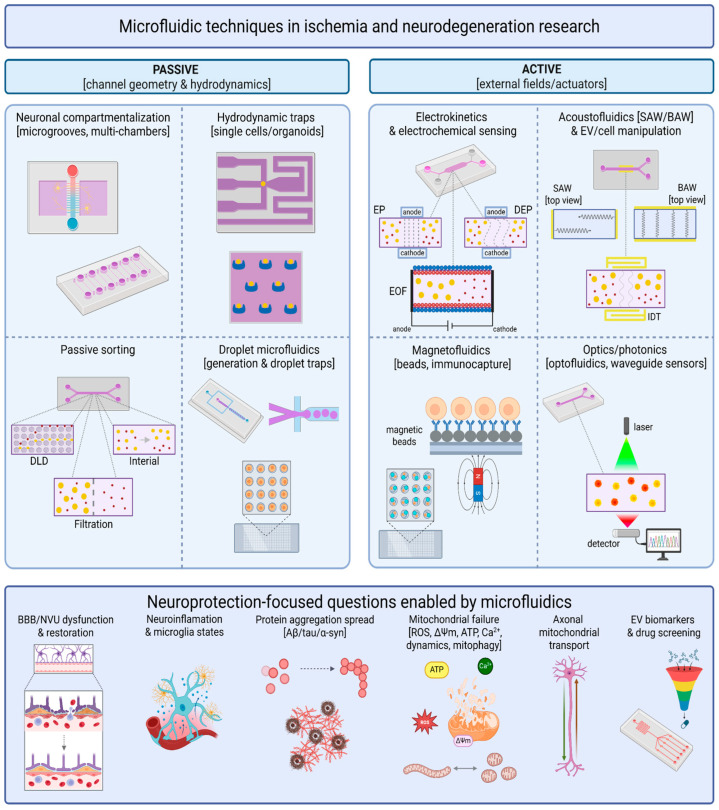
Classification of microfluidic modalities relevant to neuroprotection studies. Passive, geometry-driven modules include laminar-gradient networks, compartmentalized channels, hydrodynamic traps, microwells and label-free passive sorting; their main contribution is to define exposure history, residence time, spatial separation, wash-in/wash-out and local injury geometry. Active modules, including electrokinetic/dielectrophoretic, acoustic and magnetic operations, are shown as enabling tools for routing, enrichment, sample preparation and sensor-compatible handling. Droplet microfluidics is placed upstream because it supports dense dose mapping, aggregation-sensitive assays and counterscreens for optical or redox interference before BBB/NVU or neuronal validation. The labels denote assay functions—prescreening, exposure filtering, validation and mechanistic follow-up—rather than a technology hierarchy or equal CNS evidence across modules.

This section is organized by the uncertainty that each platform removes from compound testing (see Table 2). In practice, the most valuable microfluidic modules are those that make exposure history, injury timing, transport, or compartment-specific vulnerability experimentally explicit. For this reason, passive microfluidics—gradients, perfusion, compartmentalization, and standardized object handling—still carries the strongest decision value in neuroprotective workflows, whereas more elaborate field-driven modules are best treated as enabling technologies unless they sharpen a defined compound-testing decision—see Figure 1 [3,7,8,68,69,70].

### 4.1. Passive Microfluidics: Control by Geometry and Flow

Geometry-driven control remains the clearest example of how microfluidics improves interpretation rather than merely miniaturizing culture. Gradients, perfusion, and compartmentalization determine what a cell actually experiences in time and space: when injury begins, how quickly a compound arrives, whether washout is complete, and whether soma and axon are being challenged together or separately. In ischemia-oriented assays, this allows investigators to separate core-like and penumbra-like conditions more rigorously than in plates; in compound discovery, it makes timed wash-in, wash-out, and repeat-dose designs experimentally tractable [58,59,60,61,71].

Compartmentalized neuron chips are especially important because they convert cellular geography into a testable variable. Axons can be stressed locally, mitochondria can be perturbed in one compartment while survival is tracked in another, and neuron-glia interactions can be restored without losing spatial control. For neuroprotection, that matters because many compounds do not fail uniformly: some preserve axonal transport but not soma survival, whereas others stabilize mitochondria only in one compartment or only during a narrow time window [58,59,60,62].

Passive microfluidics also includes the less prominent but important problem of standardized object handling [86]. Hydrodynamic traps, micro-wells, and residence-time-controlled architectures do not add biological complexity, but they reduce handling variability for scarce spheroids, organoids, and cell clusters. This standardization is often more useful than adding another cell type too early, because it makes exposure and observation histories comparable across runs and across compounds [72,73,74].

### 4.2. Active Microfluidics: Control by Fields

Active-field microfluidics should be framed more cautiously. Electrokinetic, acoustic, and magnetic operations can be extremely useful for routing, concentration, enrichment, or biomarker preparation, but the direct CNS evidence that they change compound ranking remains thin. Their value is therefore mostly infrastructural: they can help prepare extracellular vesicles, enrich rare fractions, automate sample handling, or support sensor-compatible workflows, yet they are rarely the biological center of a neuroprotective assay [75,78,79,80,81,82,83,84,85].

For that reason, active modules are most persuasive when they solve a defined bottleneck. If a study needs gentle enrichment of extracellular vesicles, contactless concentration of fragile particles, or field-assisted integration with downstream sensing, these technologies are entirely appropriate. What they do not yet justify is a stronger inference that field-based manipulation alone improves the biological relevance of neuroprotective screening. In this Review, they are therefore treated as adjunct tools, not as coequal evidence domains.

### 4.3. Droplet Microfluidics as a Bridge Between Screening and Mechanism

Droplet microfluidics sits closest to chemistry and is most useful when the immediate question is whether a compound series produces a clean, interpretable signal worth escalating. Its strength lies in dense dose matrices, narrow concentration windows, kinetic perturbation, interference mapping, and efficient use of scarce material. That makes droplets particularly attractive for pleiotropic natural products, early synthetic series, and compounds whose apparent activity may depend on concentration shape rather than on a single endpoint value [51,52,53,54,55,56].

The key point is disciplined interpretation. Droplets can tell us whether a signal is concentration-dependent, whether aggregation or optical interference is likely, and whether a biochemical or short-format cellular phenotype is robust enough to deserve transport-aware follow-up. They are much less convincing as stand-alone evidence that a molecule is already neuroprotective in a CNS-relevant sense. For that reason, droplets work best as a front-end filter: they compress chemical uncertainty, and then hand only the most interpretable candidates to BBB/NVU and neuronal-chip assays.

### 4.4. Practical Constraints: Materials, Adsorption, and Solvents

Material choice is not a side issue in neuroprotective microfluidics. Many compounds of interest are lipophilic, fluorescent, redox-active, formulation-sensitive, or present only at low free concentrations, so device materials can alter the experiment before the biology begins. PDMS remains convenient for prototyping, but its tendency to absorb hydrophobic molecules and distort effective dosing is a recurring problem, especially for polyphenols and other assay-sensitive chemotypes [57,63].

The practical implication is simple: exposure should be verified rather than assumed. At minimum, studies should report device material and surface treatment, solvent limits, inlet and outlet concentrations when feasible, and whether material loss was tested for the compound family in question. Bubble control, evaporation control, and baseline osmolarity checks are similarly unglamorous but decisive, because they determine whether a platform is producing biology or artifact. In compound-centered workflows, these are part of the evidence, not merely engineering housekeeping [49,63].

## 5. BBB-on-a-Chip and Neurovascular Unit Chips as Exposure-and-Safety Filters

BBB/NVU chips currently provide the clearest decision value in microfluidic neuroprotection. Their strongest contribution is not that they fully predict clinical success; rather, they bring forward tests of exposure, efflux, barrier toxicity, endothelial activation, and vascular inflammation under flow. In practical discovery terms, they ask a narrower but decisive question: Is the candidate still usable once the barrier becomes part of the experiment [49,63,64,66,87,88,89,90]?

### 5.1. Core Design Principles and Readouts

A useful design principle for BBB/NVU work is fit-for-purpose complexity. Not every study needs the same level of biological assembly. If the goal is baseline paracellular permeability, a well-characterized endothelial barrier with appropriate support cells may be sufficient. If the question concerns neuroinflammation, post-ischemic signaling, leukocyte behavior, or disease-specific vascular dysfunction, then pericytes, astrocytes, and often microglia become mechanistic requirements rather than optional additions [41,42,49,64,68,88,89].

Readouts should follow the same logic. TEER, Papp, junctional markers, and transporter activity remain core BBB measurements, but they answer different questions and should not be treated as interchangeable. TEER is informative for ionic barrier integrity; Papp is more directly relevant to compound transport; cytokine panels, leukocyte adhesion, and endothelial activation markers become essential when the biology under study is inflammatory or vascular rather than purely permeability-based. Recent comparative analyses reinforce that model composition, flow regime, and measurement method substantially influence reported BBB performance, showing the importance of reporting discipline in interpretation [49,64].

Cell source is equally consequential. iPSC-derived BBB-like cells enable human genetics, disease-specific perturbation, and scalable access to patient-relevant material, but they do not remove the need for benchmarking. Differentiation state, transporter function, junctional maturity, and batch dependence remain real variables. The emerging 3D fully iPSC-derived BBB/NVU systems are especially promising because they combine human cell sourcing with vessel-like architecture and perfusion, but they strengthen, rather than weaken, the case for rigorous baseline validation [43,63,91,92].

### 5.2. Disease Modeling and the “BBB Filter” Concept

The most useful way to think about BBB/NVU chips in compound discovery is as an early filter rather than as a final disease surrogate. A compound may appear potent in neuron-only culture and yet fail immediately once transport, adsorption, free concentration, or barrier toxicity are measured. Conversely, compounds with primarily vascular or neuroimmune effects may look weak in neuron-only systems but gain plausibility when endothelial stress, pericyte signaling, or microglial activation are included. The BBB filter, therefore, prevents a false dichotomy between delivery and mechanism: in CNS pharmacology, transport is already part of the mechanism [42,50,63,64,65,66,67,87,88,89,93,94,95].

This logic is especially relevant for chemotypes with known exposure liabilities. Lipophilic polyphenols may lose apparent potency once adsorption and free concentration are considered; peptides and polar cargos may reveal transport failure early; and barrier-stabilizing or anti-inflammatory agents may prove more interesting once the vascular compartment is restored. Recent disease-oriented BBB-chip studies, including models tracking aggregate transport and multicellular neurovascular responses, support the idea that barrier biology can change the interpretation of an otherwise attractive compound class [66,67,90].

### 5.3. Modeling Ischemia and Reperfusion in BBB/NVU Chips

Ischemia and reperfusion are among the strongest cases for using BBB/NVU chips, because timing can be made explicit rather than approximate. Perfusate switching, OGD/reoxygenation transitions and reperfusion-linked barrier failure can be imposed with defined onset and duration, while barrier function, inflammatory signals and vascular injury are followed in parallel. This is a meaningful advance over static monocultures, where oxygen and glucose transitions are often slow, poorly resolved or inferred rather than verified [17,18,19].

At the same time, the field should not overclaim. What BBB stroke chips currently show most convincingly is that reperfusion timing, barrier dysfunction and multicellular context can materially alter assay outcome. Much less established is the stronger program-level claim that screening the same chemical series through these chips prospectively improves later translational success. For now, the evidence supports their use as timing-aware exposure and safety filters, not as validated end-to-end predictors [14,15,17,18,19].

### 5.4. From Device Physics to Deployment: What to Report

Because BBB-chip results are sensitive to flow, shear, oxygen control, materials, membrane geometry, and sensor configuration, reporting discipline is part of the biology. At minimum, studies should describe channel dimensions, flow rates, oxygen and CO_2_ control, device materials and coatings, cell sources, maturation window, TEER configuration and calibration, permeability tracers, and analytical method. Without those details, even a visually convincing barrier model remains difficult to compare across studies or across compounds [49,63,64].

A related point is benchmarking. Barrier disruptors, reference tracers, and at least one compound with known transport behavior are not optional conveniences; they are anchors for interpretation. Recent meta-analytic work has made this especially clear by showing how profoundly BBB readouts shift with model architecture, cell ratios, and shear conditions. For a compound-centered review such as this one, that is precisely why BBB/NVU chips deserve emphasis: when benchmarked properly, they expose liabilities early and in a form that can still inform medicinal chemistry or formulation decisions [49].

### 5.5. Quantifying Transport: From TEER and Papp to Exposure-Relevant Dosing

For compound triage, transport metrics should be framed around exposure rather than appearance [50,96]. TEER is valuable because it reveals ionic barrier disruption, but it does not directly quantify the amount of compound that reaches the brain-facing side. Papp is more transport-relevant, especially when measured for both the test compound and a reference paracellular tracer, yet it remains sensitive to area estimates, sampling design and material loss. In practice, TEER plus Papp plus simple mass-balance logic provides a much more honest basis for prioritization than morphology or tight-junction staining alone [14,15,50,96].

This is particularly important for lipophilic bioactives and PDMS-based systems. An apparently weak compound may simply be a poorly delivered compound. Reporting inlet versus outlet concentrations, or at least checking compound recovery under flow, can therefore prevent false-negative interpretation. In neuroprotective discovery, this matters because many promising chemotypes fail first on exposure rather than on mechanism [50,96].

Operational BBB/NVU thresholds should therefore be pre-specified locally rather than imported as universal numerical cutoffs. A decision-ready BBB/NVU filter should state, at minimum, the baseline TEER acceptance range, the allowed treatment-induced TEER change, the reference-tracer Papp range, the test-compound Papp or brain-facing recovery, and the mass-balance/recovery window used to judge material loss. These values should be benchmarked against reference tracers and at least one compound with known transport or barrier-disrupting behavior, because TEER alone cannot define a transport-relevant go/no-go decision [43,49,50,64,97].

### 5.6. iPSC-Derived BBB Models: Opportunities and Caveats

iPSC-derived BBB models deserve a prominent place in the workflow, but they still require benchmarking. Their advantages are substantial: human genetics, patient specificity, disease modeling and scalable access to otherwise difficult cell types. Yet their decision value depends on the same factors that govern other BBB systems—baseline barrier function, transporter competence, culture age and reproducibility across batches and lines [20,43,91,92].

The move toward fully iPSC-derived 3D perfused BBB/NVU models is important because it begins to close the gap between accessibility and physiological architecture. These newer systems strengthen the case for using BBB chips as mechanistic and exposure filters, particularly in disease-linked neurovascular questions. But they do not remove the need for rigorous fit-for-purpose benchmarking; rather, they make that benchmarking more urgent, because the models are becoming more capable and therefore more likely to invite overinterpretation [20].

## 6. Neuronal Injury-on-a-Chip Models for Ischemia Timing and Selective Neurodegenerative Questions

Neuronal microfluidics is most convincing where it addresses variables that plates handle poorly: localize injury, separate axons from somata, impose controlled OGD/reperfusion transitions and preserve time-resolved access to mitochondrial or transport-linked phenotypes. Those strengths matter directly for neuroprotective triage in ischemia-like injury. Their extension to Alzheimer’s disease, Parkinson’s disease and broader proteinopathies is real but selective and is strongest where chronic pathology intersects with transport stress, mitochondrial dysfunction or multicellular context rather than where the chip is treated as a generic chronic-disease surrogate [3,4,5,6,7,8,9,10,21,24,32,67].

### 6.1. Compartmentalized Neuron Culture and Axon-Targeted Injury Paradigms

Compartmentalized neuron chips remain one of the most useful microfluidic formats in neurobiology because they make neuronal polarity experimentally actionable. By separating axons from somata while preserving connectivity, they allow localized injury, spatially restricted drug delivery and direct measurement of retrograde signaling. For neuroprotection, this is especially important because axonal failure and transport defects can precede overt soma loss, meaning that survival and protection are not always the same biological event [10,21,24].

These platforms are also well-suited to mechanism testing. Axonal mitochondrial stress can be induced locally, transport tracked in real time and recovery compared after compartment-specific or global dosing. That lets the assay ask a stronger question than simple viability: does the compound preserve the vulnerable structure that fails first? In both ischemic and degenerative settings, that is often the more informative readout [21,24].

### 6.2. Implementing OGD and Ischemia-Reperfusion Dynamics on a Chip

Timed OGD and reperfusion are among the clearest cases in which chip physics changes interpretation. In conventional plate systems, solution exchange, gas transitions and reperfusion timing are often approximate. In microfluidics, they can be defined more sharply, allowing injury onset, washout, reoxygenation and delayed dosing to become explicit variables rather than hidden noise. For timing-fragile neuroprotective classes, that is not simply a technical refinement; it is a stricter test of whether the effect survives a clinically plausible window [33,34,35,58,59,60,61,62].

The real strength of these models is therefore temporal discipline. They can distinguish pretreatment-only benefit from delayed rescue, transient stabilization from durable recovery, and local stress from propagated injury. That is why neuronal OGD/reperfusion chips carry stronger decision value than many higher-complexity neurodegeneration chips: the assay logic is tighter, the failure mode is clearer, and the connection to compound triage is more immediate [33,34,35,58,59,60,61,62].

For comparison, conventional static OGD plates remain useful for inexpensive throughput and first-pass biological plausibility, but they are weaker when the decisive variable is the exact onset, duration, washout, or reperfusion-timed arrival of a compound [33,34,35,58,59,60,71]. Direct head-to-head studies showing lower false-positive rates or improved clinical enrichment remain scarce; therefore, any claim of superiority is restricted here to measurable assay variables—timing, compartmentalization, exposure history, and transport—rather than to proven downstream translational success.

### 6.3. Mitochondria-Linked Phenotypes in Microfluidic Neurons

Microfluidic neuronal models are particularly appropriate to mitochondria-linked readouts because they combine spatial control with repeated imaging under defined exposure. The most informative phenotypes are not isolated markers but coordinated behaviors: Δψm stability and recovery, ATP-linked energetic state, redox stress, mitochondrial morphology, axonal transport and, where feasible, quality-control flux. Read together, these can distinguish genuine stabilization of injury biology from movement in a single convenient marker [24,32,58,59,60,61,62].

This matters especially for pleiotropic and mitochondria-directed compounds. A molecule may transiently improve membrane potential while failing to preserve transport, or reduce ROS without improving energetic recovery. Microfluidic dosing is useful here because it turns time into a first-class variable: apparent rescue can be assessed during injury, immediately after reperfusion and at delayed intervals under the same experimental geometry. The strongest mitochondrial evidence on a chip is therefore multiparametric and time-resolved rather than marker-by-marker [24,32,58,59,60,61,62].

### 6.4. Confined Spaces and Model Validity: Lessons from SH-SY5Y and Related Systems

A recurring weakness in neuronal chip work is the assumption that geometry is biologically neutral. It is not. Confinement changes nutrient access, gas exchange, contact guidance, proliferation and baseline stress before any intended perturbation is applied. That point matters for all neuronal models, but it is especially important for widely used lines such as SH-SY5Y, where chamber size and restricted space can alter growth behavior substantially [98].

The broader implication is methodological rather than merely technical. Model validity begins before OGD, aggregation or drug dosing. If baseline physiology has already shifted because the chamber is too small or mass transport is too restricted, then the downstream neuroprotective phenotype becomes harder to interpret. In that sense, device geometry belongs in the biological methods section of the experiment, not in the invisible background [98].

### 6.5. Toward Higher Complexity: 3D Networks and Vascularized Organoids

Higher-complexity models are most persuasive when architecture itself is the question. Three-dimensional neuronal networks, organoid-on-a-chip systems and vascularized brain organoids become valuable when diffusion distance, long-term maturation, neurovascular interaction or tissue organization is mechanistically central. They are less convincing when used as default screening substrates before simpler questions of exposure, timing and material compatibility have been resolved [1,2,3,6,73].

Recent reviews support a sensible escalation logic. Organoid-on-a-chip and vascularized organoid systems are increasingly useful for maturation, tissue function and neurovascular architecture, but they remain more heterogeneous, less standardized and more demanding to benchmark than simpler neuronal chips. For a compound-centered review, that argues for restraint: use them when they answer an architecture-dependent question, not simply because they are more complex [1,2,4,6].

### 6.6. Microfluidic Models of Proteinopathy-Linked Neurodegeneration

Microfluidic neuronal networks also become relevant where neuroprotection intersects with proteostasis and pathology propagation. These systems can test whether a biochemical anti-aggregation hit still behaves coherently in living neurons with transport, compartmentalization and clearance. That is particularly relevant for tau- and α-synuclein-linked questions, where cell-free activity may not persist in a neuronal context with active trafficking and stress signaling [3,66,67].

Even here, however, the inference should remain narrow. Proteinopathy-on-a-chip systems support mechanistic intersections—propagation, trafficking, aggregate transport across the BBB, or interactions between inflammatory and aggregation stress—more convincingly than they support a universal shared assay logic across stroke, Alzheimer’s disease, Parkinson’s disease and broader proteinopathies. They are most useful when they sharpen a specific failure route, not as a rhetorical bridge between distinct diseases [3,66,67].

### 6.7. Cell-Type Diversity and Neuroimmune Integration

Neuron-only systems remain useful, but they are often not enough. Ischemic and degenerative injury is shaped by astrocytes, microglia, pericytes, endothelial signals and, in some settings, peripheral immune components. This is one of the clearest domains in which organ-on-a-chip logic outperforms simpler neuronal culture: migration, staged inflammatory exposure and neurovascular communication can be imposed directly rather than inferred after the fact [15,16,19,20].

For compound triage, the implication is important. Some candidates that look weak in neuron-only assays become more plausible once the relevant multicellular choreography is restored; others collapse once the vascular or immune context is added. That asymmetry is not a nuisance but one of the main reasons to use microfluidic systems in the first place. Their value lies in exposing context-dependent loss—or recovery—of efficacy earlier, before more expensive models are engaged.

## 7. Droplet Microfluidics: Chemistry Triage Before Transport-Aware CNS Validation

Droplet microfluidics is now a highly mature engineering platform for dense biochemical screening, compact phenotypic assays and efficient manipulation of scarce material, but its role in neuroprotection is best defined carefully. The format is often described in terms that imply miniaturization and throughput alone confer biological relevance. In practice, however, the strongest value of droplets lies earlier in the workflow. They are most useful when the question is not yet whether a compound is genuinely neuroprotective in a CNS-relevant setting, but whether it generates a signal that is chemically interpretable, concentration-dependent and worth carrying forward into more demanding models. In this view, droplet microfluidics does not compete with BBB/NVU chips or neuronal injury-on-a-chip systems. Instead, it occupies a distinct upstream position in which chemical ambiguity can be reduced before transport, multicellular context and injury timing are allowed to complicate interpretation [51,52,53,54,55,56].

This distinction is important because one of the recurrent weaknesses in neuroprotective discovery has been premature escalation. Compounds that appear attractive in simplified settings are often moved too quickly into more complex systems before their basic assay behavior has been understood. Droplets can help avoid that pattern. By making it possible to test large numbers of conditions with tight control over volume, mixing and timing, they allow researchers to determine whether a signal is stable enough to deserve scarce downstream capacity. In this sense, droplets are not a substitute for disease-relevant modeling. They are a tool for ensuring that later, biologically richer stages are reserved for compounds that have already survived a first round of chemical scrutiny [51,52,53,54,55,56].

### 7.1. Why Droplets: Scaling, Dose-Response and Single-Cell Resolution

The practical strength of droplets is not throughput alone, but the ability to obtain far more information per unit of compound, time and experimental space than would be feasible in conventional formats. Monodisperse droplet generation, precise reagent partitioning and scalable condition encoding make it possible to resolve narrow dose windows, identify non-linear or hormetic responses and examine unstable compounds under more controlled microenvironments than standard well-based assays typically allow. This matters particularly in neuroprotective discovery, where many compounds of interest are available only in limited amounts, behave unpredictably across concentration ranges, or produce apparent effects only within narrow and chemically fragile operating windows. Under those circumstances, the principal advantage of droplets is not that they are faster, but that they are more discriminating at an early stage [51,52,53,54,55,56].

A second advantage is that droplet systems can expose heterogeneity that would otherwise be flattened in bulk measurements. This is especially relevant when single-cell response distributions are likely to matter, as they often do in stress biology and early phenotypic screening. A candidate may appear moderately active in a pooled assay while in reality affecting only a small responder subpopulation, or conversely, may produce a coherent shift that is diluted by population averaging. Even when such results do not justify direct neuroprotective claims, they can still provide useful triage information by distinguishing coherent chemical behavior from sparse or noisy activity. That is often exactly the kind of information needed before a series is advanced into lower-throughput CNS-relevant assays [51,52,53,54,55,56].

More broadly, droplets help clarify a common problem in early discovery that is often poorly acknowledged: the distinction between signal abundance and signal credibility. A large number of apparent hits is not necessarily an advantage if the underlying assay landscape is full of interference, unstable concentration behavior or false-positive chemistry. Droplet microfluidics helps address this by enabling much denser interrogation of response surfaces than is usually practical in plates. In a review framed around decision value, that density matters because it helps determine not only whether a signal exists, but whether it is likely to survive the transition into more realistic biological contexts [51,52,53,54,55,56].

### 7.2. Neurorelevant Assays That Fit the Droplet Format

Not every neurobiological question belongs naturally in a droplet, and it is helpful to say so directly. The format is strongest when the assay duration is compatible with droplet stability, the readout is chemically or optically tractable, and the central aim is to determine whether a compound has a clean activity profile rather than to reconstruct a full disease state. Within those limits, several classes of neurorelevant assays map well onto droplets. Protein aggregation assays, condensate-sensitive measurements, enzyme- or redox-linked reactions, compact ROS- or Δψm-sensitive phenotypes, and digitally partitioned seeding assays all benefit from the ability of droplets to isolate reaction microenvironments and to multiply conditions with minimal material cost [51,52,53,54,55,56].

This is particularly relevant for proteinopathy-linked questions, where the distinction between direct action on the aggregation pathway and indirect suppression of a downstream reporter is often important. Recent work on α-synuclein-related systems, including digital seed amplification logic and condensate-associated aggregation assays, has strengthened the case for using microcompartmentalized formats to resolve very low-abundance or highly dynamic aggregate-linked signals [51,52,53,54,55,56]. In that setting, droplets do not replace neuronal context, but they can reveal whether there is any sufficiently robust chemistry to justify bringing the question into neurons at all. That is a more modest claim than disease modeling, but it is also a more useful one for triage.

There is also a broader conceptual point here. Many neuroprotection papers still move too quickly from “biochemical activity observed” to “therapeutic relevance implied,” even when the assay format is incapable of bearing that inference. Droplets can help prevent this slippage, but only if they are interpreted within their proper scope. Their value lies in clarifying whether the chemistry is meaningful enough to warrant escalation, not in allowing a biochemical or compact phenotypic assay to masquerade as a faithful model of disease. A review that makes this distinction explicit is likely to read as more rigorous, not less ambitious [51,52,53,54,55,56].

### 7.3. How Droplet Screens Connect to BBB/NVU Chips

The most defensible way to connect droplets with downstream CNS models is to treat each stage as answering a different class of failure question. Droplets ask whether the observed signal is real, concentration-dependent and operationally robust under chemically controlled conditions. BBB/NVU chips then ask whether that signal survives contact with transport biology, adsorption, efflux and barrier toxicity. Neuronal chips, in turn, ask whether the remaining activity persists under time-resolved injury, compartment-specific vulnerability and multicellular context. This sequential logic is more persuasive than arguing that one platform can subsume the others, because it reflects the fact that neuroprotective compounds often fail for different reasons at different stages of testing [51,52,53,54,55,56].

Seen this way, droplets function as a chemical compression step. They help narrow the set of compounds that deserve more expensive and lower-throughput investigation by removing obvious liabilities early. That role is especially valuable when dealing with chemotypes known to be vulnerable to optical artifact, aggregation, unstable dose-response behavior, or poor assay portability. Allowing such compounds to pass directly into BBB/NVU or neuronal-chip workflows without prior triage can waste both material and interpretive effort. By contrast, a disciplined droplet stage makes downstream testing more selective and therefore more meaningful.

This division of tasks also helps keep claims proportionate. A common temptation in microfluidic papers is to present every platform as part of a seamless continuum of increasing physiological realism. That language often sounds attractive, but it can blur real differences in what the assays are actually capable of demonstrating. In the present framework, droplets earn their place not by approximating disease, but by clarifying chemistry before disease-relevant constraints are imposed. That is a narrower role than full validation, but it is also one that is already well-supported by the literature and easier to defend to critical readers [51,52,53,54,55,56].

### 7.4. Droplet Assay Pitfalls and Mitigation Strategies

Like all screening formats, droplet systems introduce their own confounders, and these deserve explicit treatment rather than a passing acknowledgment. Partitioning into oil phases, surfactant effects, interface-driven enrichment, evaporation-related drift, fluorophore quenching and altered mass transfer can all distort interpretation. These problems are not unique to neuroprotective chemistry, but they are particularly relevant in this field because many compounds of interest are themselves lipophilic, fluorescent, redox-active or otherwise prone to assay interference. In other words, the chemical properties that often make a compound interesting are also the ones most likely to generate misleading behavior in droplet format when controls are insufficient [51,52,53,54,55,56].

The practical response is not to avoid droplets, but to build in orthogonal checks early. Concentration-response continuity, bulk-format confirmation, interference counterscreens, reference chemotypes and internal controls for optical or partitioning artifacts should be treated as part of the assay logic rather than as optional extras. This is especially important when the droplet stage is intended to determine which compounds deserve further investment. An upstream triage tool that fails to distinguish assay-specific behavior from chemically meaningful activity is not merely noisy; it actively distorts the downstream workflow.

At a broader level, this is also where the style of the review matters. A review that presents droplets as simple screening engines will sound technologically enthusiastic but biologically underspecified control. A review that treats them as powerful but chemically demanding tools, whose interpretive strength depends on disciplined controls, will read as more mature. For neuroprotection in particular, where false confidence has often arisen from elegant assays with underappreciated confounders, that difference in tone is important [51,52,53,54,55,56].

### 7.5. Encoding Chemical Space in Droplets and Linking to Downstream Profiling

Modern droplet microfluidics can encode chemical space far beyond simple one-compound-one-readout screening. Combinatorial mixing, barcoding, reinjection, sequential perturbation and staged droplet manipulation allow increasingly dense mapping of analog series, interaction landscapes and condition-specific activity profiles. This matters because early discovery decisions are often less about absolute potency than about the structure of uncertainty around a compound family. A series may look promising under one set of conditions, but collapse as soon as concentration spacing narrows, solvent conditions shift, or a second perturbation is added. Droplets are well-suited to making those vulnerabilities visible early [51,52,53,54,55,56].

For neuroprotective discovery, the real value of that flexibility is strategic rather than merely technical. It allows investigators to ask which liabilities are most worth falsifying first. Does the signal persist across a narrow dose grid? Does a putative hit remain active when the optical readout changes? Does a combination profile suggest genuine complementarity or only an overlapping assay artifact? These are exactly the kinds of questions that should be resolved before more physiologically elaborate systems are engaged. In this sense, droplets do not simply accelerate screening; they sharpen prioritization.

That logic also fits naturally with the overall architecture of this Review. Chemistry should not be treated as a preliminary hurdle that is quickly forgotten once a compound enters a chip-based biological model. It should remain visible as a source of potential failure throughout the workflow. Droplets make that possible by giving chemistry its own rigorous stage before transport and context are added. That is one of the strongest reasons to keep them in the framework, provided their role is carefully defined and not overstated [51,52,53,54,55,56].

## 8. Mitochondria-Centered Readouts and Selected Adjunct Instrumentation

Mitochondria occupy a particularly important place in neuroprotective discovery because they sit near the intersection of energetic collapse, ROS imbalance, calcium dysregulation, impaired transport and delayed cell loss. In both ischemic and neurodegenerative settings, they often function less as one pathway among many than as a convergence point through which several forms of stress become biologically consequential. At the same time, mitochondrial phenotyping is easy to overuse. A long list of mitochondrial endpoints can create the appearance of mechanistic depth without necessarily clarifying whether a compound should advance. For that reason, the value of mitochondria-centered readouts depends on whether they change a decision rather than simply multiply observations [60,72,74].

**Figure 2:**
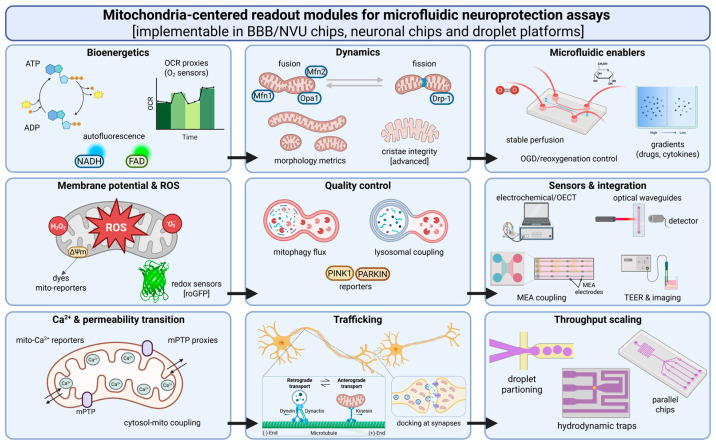
Mitochondria-centered readouts in microfluidic neuroprotection workflows. Membrane potential and ATP/energy status test energetic rescue; ROS/redox and Ca^2+^ dynamics assess stress signaling and excitotoxic coupling; morphology, fusion-fission balance, mitophagy flux and axonal mitochondrial transport test whether recovery is durable and compartment specific. Readout depth should match the workflow stage: compact prescreen may use a small, interference-controlled signal, whereas neuronal OGD/reperfusion chips can justify time-resolved combinations of Δψm, ROS, ATP, transport and structural endpoints. Calibration, phototoxicity checks, dye-loading controls and orthogonal validation are especially important for redox-active or fluorescent compounds.

That principle is especially important in microfluidic systems, where repeated imaging, defined exposure histories and spatial compartmentalization make mitochondrial measurements unusually attractive. These features can generate highly informative data, but they can also encourage overinterpretation if every detectable mitochondrial signal is treated as evidence of rescue. A more disciplined approach is to ask which mitochondrial readouts are most relevant at each platform stage and which combinations of markers actually strengthen inference. Within that logic, the most useful measurements are those that connect mitochondrial state to transport, survival, barrier integrity, inflammatory context or structural preservation (see Figure 2). Readouts that remain isolated from those larger questions are much less valuable, no matter how technically elegant they appear [60,72,74].

### 8.1. Core Mitochondrial Readouts Relevant to Ischemia and Neurodegeneration

Membrane potential remains one of the most sensitive and informative early signals because it integrates respiratory function, ion homeostasis and impending energetic failure. In models of ischemia-like injury, collapse of Δψm often marks the transition from reversible stress to deeper bioenergetic compromise, while in chronic neurodegeneration, altered membrane potential dynamics may reveal sustained mitochondrial vulnerability even before overt loss of viability. ATP-linked measurements add an important complementary layer by providing a more functional readout of whether apparent mitochondrial rescue translates into preserved energetic competence. Interpreted together, Δψm and ATP can help distinguish transient stabilization from more substantive recovery [60,72,74].

ROS-sensitive assays remain equally important, but they are also among the most vulnerable to distortion. This is especially true in microfluidics, where repeated imaging, low working volumes and compound-dependent optical effects can magnify both genuine signal and measurement artifact. For that reason, ROS readouts are most useful when paired with calibration logic and, where possible, orthogonal checks that separate biological rescue from dye-specific behavior. On their own, they can be suggestive; in context, they can become genuinely decision-relevant [60,72,74].

Mitochondrial morphology, dynamics and quality-control pathways increasingly deserve inclusion because they help answer a different question: not whether mitochondria are stressed, but whether they are recovering in a way that is likely to endure. Fusion-fission balance, fragmentation patterns and mitophagy competence all shape whether a cell can restore mitochondrial function after an insult or merely delay the progression toward failure. These are therefore not secondary mechanistic details. In the context of neuroprotective triage, they are most persuasive when they are linked to axonal transport, barrier function, survival or structural preservation rather than treated as standalone signatures of sophistication [60,72,74].

### 8.2. Integrated Sensing and “Instrumented” Chips

Integrated sensing becomes genuinely valuable when it verifies the perturbation itself rather than simply increasing the number of endpoints measured. Continuous on-chip monitoring of barrier-relevant and metabolic parameters, including TEER, can establish when a stressor began, whether it reached the intended magnitude and how recovery unfolded over time. That is particularly important in ischemia-mimetic and barrier-relevant systems, where the nominal protocol may not accurately reflect what the cells experienced. In such cases, an embedded sensor does not merely enrich the dataset; it reduces uncertainty about the underlying experiment [73,97].

This distinction matters because sensor integration is sometimes presented as a technological advance in its own right, independent of its biological contributions. A more convincing interpretation is that instrumented chips are useful when they constrain hidden variables that would otherwise weaken downstream claims. If key stress conditions are assumed rather than verified, even a sophisticated rescue phenotype remains more fragile than it appears. By contrast, when sensing confirms the timing and magnitude of the underlying perturbation, the biological interpretation becomes more secure.

For neuroprotective discovery, this is especially relevant because many compounds fail not only because they lack a mechanism, but because the assay environment is less controlled than the figures imply. Sensor-integrated chips, therefore, deserve emphasis not because they make experiments look more advanced, but because they can make them more trustworthy. This is fully consistent with the broader logic of the Review, which places the greatest value on platforms that remove uncertainty from compound decisions rather than merely adding complexity [73,97].

### 8.3. Extracellular Vesicles as Mitochondria- and Neuroinflammation-Linked Biomarkers

Extracellular vesicle workflows can add a useful translational layer to chip-based studies, but only when their role is defined carefully. In the present framework, EVs are most relevant when they connect an on-a-chip perturbation to a portable biomarker logic—for example, by capturing vesicle populations of interest and enabling downstream analysis of cargo changes in a form that might later translate beyond the chip itself [76,77,99]. Used in that way, EV analysis can extend the interpretive range of microfluidic assays without requiring that every chip become a full biomarker platform from the outset.

This selective approach is important because EV biology is easy to overextend rhetorically. The fact that vesicles can carry mechanistically interesting cargo does not automatically make them useful in every screening context. Their strongest value in neuroprotective discovery lies in answering a question the chip itself cannot answer clearly: whether a cellular response leaves a measurable extracellular trace that is potentially compatible with translational follow-up. In that role, EV workflows can strengthen the bridge between mechanistic screening and later deployment.

The recent literature supports both sides of this balance. On the one hand, microfluidic enrichment methods are becoming increasingly capable of isolating vesicle populations from limited samples. On the other hand, the biological interpretation of EV cargo still depends heavily on context, enrichment logic and analytic rigor. A review that includes EVs as an adjunct, rather than a universal requirement, is therefore likely to seem more grounded and more consistent with the overall staged logic of the manuscript [77,99].

### 8.4. Matching Readouts to Platform Stage

One of the central interpretive principles of a staged workflow is that not every platform should attempt to measure everything. In early droplet or compact prescreen formats, a small set of chemically informative readouts—such as redox-sensitive responses or compact Δψm-linked phenotypes—may be sufficient to eliminate fragile or misleading candidates. In BBB/NVU filters, mitochondrial signals become more persuasive when interpreted together with transport, barrier integrity and endothelial stress. In neuronal injury chips, the richest and most useful phenotype sets are usually those that are explicitly time-resolved and multiparametric, combining membrane potential, energetic recovery, transport competence and survival or structural preservation (see Table 3) [60,72,74,97].

This matching of readouts to the platform stage is not merely an issue of efficiency. It is central to interpretability. A workflow in which each stage has an appropriate depth of measurement is much easier to defend than one in which every stage accumulates a broad but poorly prioritized marker set. In the latter case, the paper may appear rich in data while remaining weak in decision value. In the former, the measurements remain visibly connected to progression through the pipeline.

That is particularly important for mitochondrial biology, which is both biologically central and experimentally attractive. Because mitochondrial phenotypes are so informative, there is a temptation to turn them into an endlessly expanding panel. A more convincing strategy is to use them selectively, at the point where they most meaningfully sharpen triage. That keeps mitochondrial biology in the workflow as a genuine decision tool rather than as a source of mechanistic ornamentation [60,65,73,97].

### 8.5. Calibration and Assay Controls for Mitochondria-Linked Dyes and Reporters

Mitochondria-linked dyes and reporters are among the most useful tools in this space, but they are also among the most fragile. Signal intensity can change with dye loading, illumination history, membrane permeability, intracellular compartment volume and direct optical interaction with the compound under study. In microfluidic systems, where low volumes and repeated imaging often increase sensitivity, these vulnerabilities become even more consequential. The practical implication is that calibration and counter-control logic should be explicit rather than assumed [60].

For Δψm-sensitive probes, collapse controls, and recovery references remain essential. For ROS-linked dyes, orthogonal validation is particularly important whenever the compound class itself is redox-active or fluorescent. These are not merely good laboratory habits; they are the conditions under which a mitochondrial signal becomes interpretable as biology rather than assay chemistry. This is especially relevant for polyphenols and other pleiotropic bioactives, whose apparent mitochondrial benefit can diminish sharply once autofluorescence, quenching or concentration-dependent partitioning are controlled [60].

### 8.6. Coupling Mitochondrial Readouts to Barrier and Inflammatory Phenotypes

Mitochondria do not function independently of vascular and inflammatory biology, and one of the advantages of microfluidic systems is that they allow those connections to be studied rather than abstractly asserted. Cytokine signaling, endothelial stress, altered transport and microglial activity can all change whether a mitochondrial phenotype is best interpreted as rescue, delay or artifact. This is why the most convincing chip studies do not ask simply whether mitochondria changed, but whether mitochondrial stabilization is coherent with barrier protection, inflammatory modulation or structural preservation [41,42,49,63,64,65].

That integrative logic is particularly important in neuroprotection, where improvements in a single mitochondrial marker can easily create a misleading impression of therapeutic promise. A compound that transiently stabilizes membrane potential may not be meaningfully protective if it fails to preserve barrier function or worsens inflammatory stress. By contrast, when mitochondrial rescue aligns with improved transport, reduced barrier liability or preserved neuronal structure, the inference becomes much stronger.

This is one of the clearest reasons why microfluidic systems matter in this field. They allow mitochondrial biology to be interpreted in context rather than as a disconnected biomarker [41,42,49,63,64,65].

## 9. A Staged Screening Framework for Compound Triage

The staged framework proposed here should be understood as a within-program triage logic rather than as a universally validated algorithm. Its purpose is not to prescribe a single path for every neuroprotective campaign, but to make recurrent failure routes visible earlier and in a more structured way. These recurrent routes include chemical fragility, transport failure, barrier liability, timing mismatch and loss of efficacy once multicellular context is restored. By organizing the workflow around these failure modes, the framework shifts attention from platform novelty to decision value. It asks which uncertainty should be removed first for a given class of compounds [34,35,49,50,58,59,60,63,64,72,73,97].

This emphasis is important because neuroprotective discovery has historically suffered from a mismatch between assay ambition and assay order. Compounds are often moved into biologically complex systems before the most basic interpretive uncertainties have been resolved, or conversely filtered out too early on the basis of models that fail to capture the failure route most likely to matter. A staged approach is meant to correct that sequencing problem. It does so by aligning the logic of the assay with the logic of the compound class under study.

**Figure 3:**
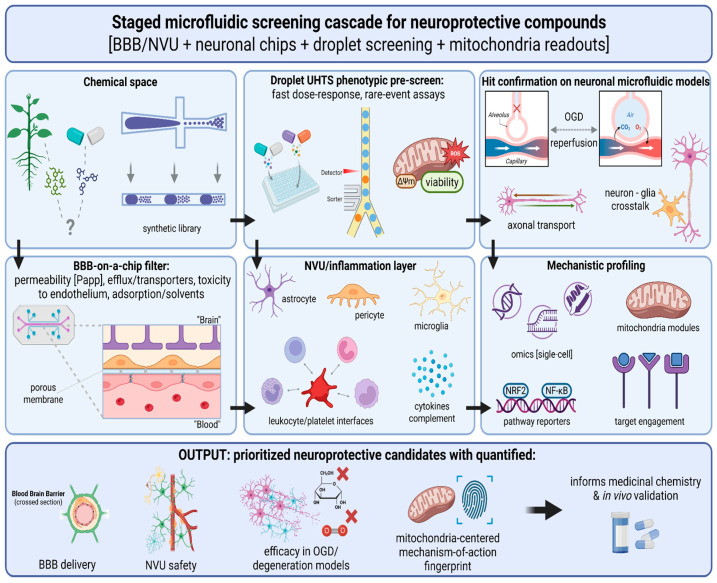
Illustrative within-program staged compound-triage framework linking chemistry-aware prescreens, BBB/NVU filters, neuronal OGD validation and mechanistic profiling. The figure is a decision map, not a fixed pipeline or prospectively validated standard. Stage 0 defines chemotype-specific liabilities, including solubility, stability, optical interference, adsorption, redox activity, vehicle tolerance and likely BBB exposure constraints. Stage 1 tests whether the signal is reproducible, concentration-dependent and free from obvious assay artifacts. Stage 2 uses TEER, Papp, compound recovery, endothelial activation and inflammatory readouts as exposure-and-safety filters. Stage 3 asks whether activity persists under timed OGD/reperfusion, delayed dosing and compartment-specific vulnerability. Stage 4 integrates mitochondrial, barrier, inflammatory and functional readouts to judge whether a coherent development hypothesis remains. The arrows indicate information flow and staged escalation, not a mandatory linear sequence.

The framework is therefore intentionally operational. It is not trying to settle which biological model is most realistic in the abstract. Instead, it identifies which type of model is most informative at each step of triage (see Figure 3) [34,35,49,50,58,59,60,63,64,72,73,97].

### 9.1. Stage 0: Chemistry Space and Assay-Aware Library Design

Stage 0 begins before any chip experiment is performed, because many downstream failures are already predictable from chemistry. Radical scavengers are typically constrained by timing and therefore require reperfusion-aware logic from the outset. Polyphenols and other pleiotropic natural products need explicit checks for solubility, adsorption and optical interference before any biological interpretation becomes persuasive. Peptides and polar cargos need early exposure realism, while mitochondria-directed agents require assays that can distinguish transient signal improvement from durable functional rescue. These differences mean that platform choice should not begin from the generic question of what is available, but from the more specific question of what is most likely to falsify the dominant liability of the compound class [51,52,53,54,55,56,57].

This stage is often omitted in practice because it can appear less biologically interesting than later cell-based experiments. Yet from a discovery standpoint, it may be one of the most important stages in the entire workflow. A chemically fragile series can consume substantial downstream effort before its limitations become obvious, especially when early assays are chosen for convenience rather than interpretive fit. Stage 0 is therefore not a formality. It is where the workflow becomes compound-aware.

There is also a conceptual advantage to making this stage explicit. It prevents the review from implying that microfluidics begins only once cells enter channels. In reality, much of the value of a microfluidic workflow depends on whether chemistry and assay design were aligned before that point. A paper that acknowledges this openly usually reads as more rigorous and more practically useful [51,52,53,54,55,56,57].

### 9.2. Stage 1: Ultrahigh-Throughput Droplet Prescreen

Stage 1 is designed to remove compounds that are attractive on paper but operationally fragile in practice. Droplet assays are particularly well-suited to this task because they can expose concentration dependence, aggregation liability, optical interference and response heterogeneity while consuming minimal material. For many chemical series, that information is more valuable at the beginning than immediate progression into complex chip models whose own assay behavior may still be uncertain [51,52,53,54,55,56].

This stage is especially useful for compounds with known assay liabilities or poorly behaved SAR patterns. A dense droplet-based prescreen can determine whether the observed activity is continuous or erratic, whether apparent hits depend on narrow and unstable conditions, and whether the chemical signal is robust enough to justify more resource-intensive follow-up. In that sense, Stage 1 is not simply a filter for weak compounds. It also serves as a filter for ambiguous compounds.

Importantly, a compound that fails at this stage does not necessarily fail because it lacks biological interest. It may fail because the available chemistry is not yet assayable in a trustworthy way. That distinction matters in a triage framework because it allows chemistry problems to be recognized as chemistry problems rather than being misread later as failures of biology. That alone can make the overall workflow much more rational [51,52,53,54,55,56].

### 9.3. Stage 2: BBB/NVU Filter for Delivery and Safety

Stage 2 asks whether the remaining chemistry survives contact with transport biology. This is often decisive for lipophilic natural products, peptides, barrier-active compounds and any class in which delivery, efflux or endothelial stress is likely to shape apparent activity. The goal here is not to simulate the full disease state, but to determine whether permeability, adsorption, barrier toxicity or endothelial activation fundamentally alter compound plausibility before more elaborate neuronal validation is attempted [49,50,63,64,97].

This stage has unusual practical importance because it prevents a common error in neuroprotective discovery: treating transport as a downstream concern rather than as part of the mechanism itself. In CNS pharmacology, a compound that cannot reach the relevant compartment under realistic conditions is not simply incompletely characterized; it is already compromised as a candidate. BBB/NVU chips make that liability visible earlier, while there is still room for medicinal chemistry, formulation change or strategic de-prioritization.

Stage 2 also prevents an opposite form of misinterpretation. Some compounds that appear weak in neuron-only systems become more plausible once a barrier, vascular or neuroimmune context is introduced. That asymmetry is one of the strongest arguments for using BBB/NVU chips as filters rather than as optional later refinements. They do not merely exclude false positives; they can also rescue compounds that would otherwise be dismissed for the wrong reason [49,50,63,64,97].

### 9.4. Stage 3: Neuronal Chip Validation Under OGD and Reperfusion Dynamics

Stage 3 asks whether the benefit survives clinically meaningful timing and selective vulnerability. At this point, the workflow should no longer be testing whether the chemistry is merely active, but whether the observed activity remains visible when injury timing, delayed dosing and compartment-specific neuronal stress are made explicit. Neuronal microfluidics is particularly strong at this stage because it can impose OGD, reperfusion and localized injury with far greater temporal and spatial discipline than conventional plate assays [33,34,35,58,59,60,61,62].

This is also the point at which the distinction between pretreatment-only benefit and true rescue becomes especially important. Many compounds that look promising in simplified models prove to depend on prophylactic or tightly timed dosing windows that are difficult to justify in clinically realistic contexts. Microfluidic neuronal systems are valuable because they can expose that dependence rather than masking it under broad protocol averages. They allow the workflow to ask not simply whether a compound works, but when it works and for which vulnerable structure.

More broadly, Stage 3 is where the concept of neuroprotection becomes more precise. Protection may involve survival, axonal preservation, transport competence, mitochondrial recovery or delayed structural rescue, and these are not always equivalent. A properly designed neuronal-chip stage can make those differences visible. That is one reason why it deserves a distinct place in the framework rather than being folded into generic “advanced cell testing” [33,34,35,58,59,60,61,62].

### 9.5. Stage 4: Mechanistic Profiling and Triangulation

Stage 4 is not included to reward complexity for its own sake. Its purpose is to test mechanistic coherence. If a compound improves one readout but fails on barrier function, mitochondrial recovery, inflammatory context or structural preservation, that inconsistency is itself informative. In a staged workflow, such mismatches are not inconveniences to be smoothed away; they are often the clearest indication that apparent protection is conditional, context-dependent or assay-specific [41,42,49,63,64,65].

This is where triangulation becomes valuable. A compound that remains favorable across transport-aware, time-resolved and mechanistically linked readouts is much more persuasive than one that excels only in a single assay domain. Conversely, a compound that collapses under triangulation may still reveal important insights into the biology of the system or the limitations of the assay. Stage 4, therefore, functions both as a confirmation step and as a final interpretive stress test [41,42,49,63,64,65].

### 9.6. Decision Points and Reporting Checklists

A staged framework only helps if each stage has explicit exit rules (see Table 4). That means pre-specifying what constitutes advancement, what constitutes failure and what triggers repeat testing or orthogonal confirmation. Without such rules, a pipeline can become descriptive rather than selective, with compounds drifting forward because no formal reason has been given to stop them. Recent fit-for-purpose and organ-chip guidance strongly supports this kind of disciplined pre-specification, particularly in complex microphysiological systems where reproducibility depends as much on quality management as on biological design [43,49,50,64].

This point is easy to underestimate because reporting checklists can seem bureaucratic when compared with more mechanistic sections of a review. In practice, however, they are central to interpretability. A complex assay without pre-defined decision logic is often less informative than a simpler assay with transparent advancement criteria. For neuroprotective triage, that matters because much of the historical difficulty in the field has come from uncertainty about what a positive preclinical signal was actually allowed to mean [43,49,50,64].

### 9.7. Timing as a Design Variable

One of the clearest practical advantages of microfluidics is that it turns therapeutic timing into a controllable experimental variable rather than a loosely defined protocol detail. Dosing can be applied as pretreatment, during injury, at the onset of reperfusion or after precisely defined delays, while exposure remains spatially and temporally controlled. This is particularly important in neuroprotection, where many apparently strong compounds lose efficacy when moved from prophylactic designs to more clinically plausible delayed-treatment conditions.

Treating timing as a design variable rather than as background noise changes the character of the workflow. It forces the assay to confront one of the dominant historical liabilities of the field: the mismatch between experimental intervention windows and therapeutic reality. A compound that survives this transition deserves more confidence than one whose benefit disappears as soon as timing becomes realistic. In this sense, temporal discipline is not a refinement at the edge of the workflow. It is one of the central reasons to use microfluidic systems at all.

### 9.8. Comparative Decision Examples and Limits of Evidence

Few studies have prospectively tested an end-to-end microfluidic workflow in which neuroprotective compounds are re-ranked against a conventional screening pipeline. The examples presented in Table 5 should therefore be interpreted as evidence-anchored decision examples and comparative benchmarks, not as proof that microfluidic workflows already improve clinical outcomes. Their value is more specific: they illustrate which uncertainty a given microfluidic stage can expose earlier than a standard static plate assay, cell-free assay or neuron-only culture. In this sense, the examples support the logic of staged triage without overstating the current level of predictive validation.

Table 5 anchors the proposed framework in existing studies that correspond to different decision points in the workflow. Wu et al. provide a BBB-chip benchmark for ischemia-reperfusion injury, showing how barrier dysfunction and reperfusion-linked responses can be assessed under controlled flow [35]. Pediaditakis et al. demonstrate a human Brain-Chip neuroinflammation model in which TNF-α exposure links BBB permeability, glial activation and cytokine readouts [42]. Qiu et al. extend this logic toward a perfusable 3D human NVU platform suitable for assessing brain drug delivery and immune-cell extravasation [63]. Vroman et al. use compartmentalized human dopaminergic neurons to distinguish routes of α-synuclein propagation, thereby illustrating how microfluidics can resolve compartment-specific mechanisms [67]. Finally, MacKerron et al. provide a perfusion-based calcium-imaging benchmark for testing CNS-active compounds [100]. Together, these studies show how microfluidic systems can clarify exposure, timing, barrier, inflammatory and compartment-specific uncertainties, while still falling short of proving clinical predictivity [35,42,63,67,100].

These examples also clarify how the same staged logic can be applied to different compound classes. For NXY-059-like radical scavengers, the proposed workflow would not retrospectively claim that a chip could have predicted clinical failure. Instead, it would ask whether apparent activity survives post-insult or reperfusion-timed dosing and whether measurable brain-facing exposure is achieved. For edaravone-like candidates, the key question would be whether the benefit persists across a defined reperfusion window rather than only under pretreatment conditions. For polyphenols and other pleiotropic natural products, the first decision is often whether the signal remains interpretable after adsorption, solubility, autofluorescence and quenching controls. For mitochondria-directed compounds, the critical issue is whether survival, Δψm, ATP-linked recovery and axonal mitochondrial transport move coherently after delayed dosing. For proteinopathy-oriented modulators, droplet aggregation assays and compartmentalized neuronal chips answer different questions: the former test biochemical or aggregation-sensitive activity, whereas the latter test whether this activity remains meaningful in living neurons with transport, compartmentalization and clearance. These examples, therefore, make the framework more operational: each platform is useful only when it removes a defined uncertainty that matters for the compound class under consideration.

## 10. Operational Guidance and Limits of Standardization

The confounders discussed throughout this Review are well-documented, but universal numerical thresholds are not. For that reason, the goal of this section is not to provide rigid rules. It is to articulate a level of operational discipline that allows within-program comparability and reduces the risk of overinterpreting technically impressive but poorly benchmarked systems. Recent organ-chip guidance, fit-for-purpose recommendations and translatability reviews converge on a similar conclusion: stronger inference comes less from maximal complexity than from clearer quality control, benchmark logic and reporting discipline [43,49,50,64]. Accordingly, this Review draws on fit-for-purpose MPS guidance and organ-on-chip implementation caveats to support a reproducibility-focused approach to reporting [101,102].

This point is especially relevant in neuroprotective microfluidics, where biological and engineering uncertainties are often entangled. A weakly controlled device can create the illusion of biology, while a biologically interesting effect can be obscured by unnoticed operational drift. Standardization, in this context, does not mean forcing every system into one universal format. It means defining the minimum level of transparency and internal consistency needed for results to support compound decisions.

That distinction is worth making explicitly, because calls for standardization can otherwise sound either too simple or too rigid. What the field needs is not less methodological creativity, but a more disciplined relationship between innovation and interpretability. That is the spirit in which the following points are best read [43,49,50,64].

Three standardization questions are especially important before these workflows can scale: (i) whether nominally identical devices produce comparable shear, residence time, oxygen delivery and adsorption profiles; (ii) whether biological baselines remain stable across fabrication lots, differentiation batches and operators; and (iii) whether the readout and analysis pipeline can handle enough independent devices to support compound ranking rather than single-device proof-of-concept demonstrations. Inter-laboratory transfer, device-to-device tolerances, and automation of fluid handling and image analysis should therefore be treated as translational bottlenecks, not as peripheral engineering details [49,64,101,102].

### 10.1. Device Material Selection and Compound Adsorption

Material choice belongs at the center of discovery-facing microfluidics because polymer-dependent compound loss can be large enough to alter apparent potency in biologically meaningful ways. This is particularly true for hydrophobic molecules, low free concentrations and chemotypes that already operate close to the threshold of detectable efficacy. Recent comparative studies reinforce that sorption differs substantially across materials and may persist into washout, meaning that actual exposure history is often much less obvious than nominal dosing suggests [57].

This has direct consequences for neuroprotective interpretation. A compound that appears weak in a microfluidic device may not be biologically inactive at all; it may simply be poorly delivered because the system removes or delays access to the relevant free concentration. Conversely, apparent delayed effects can sometimes reflect release behavior from the device rather than the biology of the cells. These are not peripheral technicalities. They are mechanisms by which a platform can distort the compound decision itself.

For that reason, device material should be treated as part of the biological methods of the experiment, not as a purely engineering footnote. This point deserves prominence because it affects the reliability of every downstream stage [57].

### 10.2. Oxygen and Nutrient Control: Validating “OGD” in Microdevices

In OGD or ischemia-mimetic experiments, intended oxygen and glucose conditions should be verified rather than inferred from nominal protocol design. Microdevices can generate unexpected gradients because geometry, gas permeability, flow and local consumption are tightly coupled. As a result, the actual perturbation experienced by the cells may differ substantially from what the protocol description implies. This is especially important in low-volume systems, where minor design features can have disproportionate consequences [35,73].

Where possible, on-chip verification of oxygen delivery and related stress conditions substantially strengthens interpretation because it confirms that the intended stress occurred, when it began and how it resolved. This does not mean that every study must become heavily instrumented. It means that the more central OGD timing is to the biological claim, the harder it becomes to justify leaving it unverified. That principle is fully consistent with the broader argument of the manuscript: the most useful technologies are those that reduce uncertainty about what the compound and the cells actually experienced [35,73].

### 10.3. Barrier Measurements: TEER Is Necessary but Not Sufficient

TEER remains an important barrier metric, but it should not be asked to bear more interpretive weight than it can support. Because it reports ionic conductance, TEER can shift with electrode geometry, temperature, medium composition and measurement configuration even when compound transport is largely unchanged. For discovery-facing work, this means that TEER is most informative when paired with permeability, mass-balance or recovery logic rather than treated as a stand-alone proxy for successful delivery [43,49,50,97].

This distinction is particularly important in papers that aim to prioritize compounds. A visually convincing barrier or a high TEER value can create a strong impression of physiological quality, but neither directly addresses how much compound reaches the brain-facing compartment. In the context of triage, transport relevance matters more than barrier appearance alone. A compound may pass through a tight barrier poorly, or through a modest barrier efficiently; TEER on its own cannot resolve that distinction [43,49,50,97].

### 10.4. Throughput Realism: Aligning Ambitions with Bottlenecks

One of the common rhetorical excesses in microfluidics literature is the tendency to speak about throughput in device terms while ignoring the actual bottleneck of the workflow. Droplet generation may be extremely fast, but imaging, segmentation, long-term culture, cell differentiation or off-chip analysis may still dominate the real timeline. A platform, therefore, becomes useful not when one component is ultrafast in isolation, but when overall throughput and interpretability are honestly aligned [51,52,53,54,55,56].

This matters because exaggerated throughput claims often create unrealistic expectations about scalability and deployment. In neuroprotective screening, where the most informative assays may involve complex cell states or time-resolved injury paradigms, nominal chip speed is only one part of the operational picture. A more persuasive approach is to identify where throughput genuinely reduces uncertainty per unit effort and where it merely shifts the bottleneck elsewhere.

### 10.5. Statistical Design and Batch Effects

Microfluidic assays are highly susceptible to batch effects arising from fabrication variability, differentiation batches, media preparation, coating stability and day-to-day flow conditions. Because these variables are distributed across both engineering and biology, they can be difficult to identify retrospectively if the replication structure has not been designed carefully. This is one reason why recent fit-for-purpose guidance has become so important for MPS work [43,49,50,64].

In practical terms, the most persuasive studies make batch structure visible. They report biological replicates, device replicates, fabrication or differentiation batches, and pre-defined control anchors rather than relying on visually compelling single runs. This does not eliminate variability, but it turns it into something that can be interpreted rather than hidden. For a staged screening framework, this is essential because progression decisions are meaningful only if the assay is stable enough for failure and success to mean the same thing across runs [43,49,50,64].

### 10.6. Bridging Physiology and Screening: Interpretability as a Design Goal

Physiological realism is useful only when it remains interpretable. A smaller set of mechanism-anchored readouts, measured consistently across stages, is usually more valuable than a broad panel that changes unpredictably from platform to platform. Recent organ-chip guidance is particularly helpful on this point because it encourages fit-for-purpose design rather than reflexive escalation of complexity [43,49,50,64].

This is highly relevant to neuroprotection, where there is often a temptation to add more cell types, more sensors and more endpoints in the hope that realism will automatically improve inference. Sometimes it does. Just as often, it obscures the decision that the assay was meant to support. A platform with moderate complexity and strong interpretive anchors is usually more useful than a highly elaborate system whose readouts are difficult to integrate.

For that reason, interpretability should be treated as an explicit design goal rather than as a by-product of good engineering.

### 10.7. When to Move Beyond 2D: Decision Rules for 3D and Vascularized Models

Escalation to 3D, organoid-associated or vascularized models should be justified by a specific decision need rather than by a general preference for complexity. Moving beyond simpler formats is worthwhile when diffusion distance, tissue organization, long-term maturation or neurovascular architecture is central to the biological question. It is much less compelling when the main unresolved uncertainty still concerns chemistry, exposure or timing. In that sense, complexity should be earned by the decision being asked, not by the availability of a more elaborate platform [1,2,6,73].

This principle is helpful because it provides a rational basis for escalation. A workflow that starts simple and becomes more complex only when a clear interpretive benefit is expected is easier to defend than one that defaults immediately to organoid-on-a-chip or vascularized systems. It also helps preserve scarce experimental capacity for the questions that truly require architectural richness.

### 10.8. Reporting Checklist: A Minimal Set

At minimum, discovery-facing studies should report device material, relevant dimensions, flow conditions, cell source, maturation state, dosing scheme, exposure controls and the logic of benchmark compounds or calibration anchors. This is not an administrative detail. It is the minimum information needed to decide whether a result is interpretable across compounds and across iterations of the same workflow. Recent fit-for-purpose and guide-style papers make this point very clearly [43,49,50,64].

The importance of such reporting becomes even greater when a paper aims to support compound triage rather than merely present a proof-of-concept platform. In that setting, missing operational detail is not just an inconvenience for reproducibility. It weakens the meaning of the biological claim itself. A compound cannot be said to have survived a given filter if the conditions of that filter are insufficiently described.

### 10.9. Operational Confounders and Benchmark Controls

Operational rigor becomes most visible when experiments fail for reasons unrelated to intended biology: bubbles, leaks, coating drift, unstable sensors, unverified oxygen transitions or compound loss to materials. These are familiar problems to anyone working hands-on with microfluidics, yet they are still too often treated in the literature as unfortunate but secondary nuisances. In a discovery-facing framework, they should be treated differently. They are alternative explanations for apparent biology and therefore belong within the interpretive structure of the workflow [43,49,50,57,97].

The strongest response is not to pretend such confounders can always be eliminated, but to use benchmark controls that reveal them early. Reference tracers, exposure recovery checks, positive and negative control compounds, and calibration routines for key sensors all help distinguish true biological variance from operational failure. These controls do not make a system less innovative. On the contrary, they are what allow innovation to become trustworthy.

The operational considerations discussed in Section 10 are consolidated in Table 6 as a minimal reporting and quality-control checklist intended for within-program use rather than as a field-wide standard.

## 11. Conclusions

Microfluidic systems should not be presented as miniature substitutes for stroke or neurodegenerative disease, and the current evidence does not justify treating them as validated predictors of clinical neuroprotection. A more defensible and useful conclusion: microfluidic platforms can make recurrent sources of compound failure visible earlier, under more controlled exposure, timing and multicellular conditions than many conventional workflows allow.

These recurrent liabilities include chemical fragility, poor assayability, compound adsorption, optical artifact, insufficient BBB/NVU exposure, barrier toxicity, narrow treatment windows and loss of efficacy once vascular, glial or inflammatory context is restored. The strongest role of microfluidics in this Review is therefore not to imitate disease in miniature, but to support structured compound triage. In that role, each platform is valuable when it removes a specific uncertainty: whether the signal is chemically reliable, whether transport and barrier safety are acceptable, whether benefit persists under reperfusion-relevant timing and whether mechanistic readouts remain coherent across neuronal, mitochondrial and neurovascular endpoints.

This evidence is not uniform across all platform classes. BBB/NVU chips currently provide the strongest support as exposure- and safety-aware filters, particularly when transport, endothelial stress, and inflammatory context materially shape interpretation. Compartmentalized neuronal microfluidic models are most convincing as timing-sensitive validation systems, especially under OGD/reperfusion logic and in settings where selective vulnerability, axonal stress or mitochondrial recovery matter to the compound decision. By contrast, droplet microfluidics contributes most clearly upstream, where it reduces chemical ambiguity through dense dose mapping, aggregation-sensitive assays and counterscreens for interference. Taken together, these observations argue against treating all microfluidic formats as interchangeable and instead support a staged, question-driven utilization of different platforms according to the dominant uncertainty they are best able to resolve.

A second conclusion is that compound class matters as much as platform choice. Radical scavengers, pleiotropic natural products, barrier-active agents, neuroimmune modulators and mitochondria-directed compounds fail for different reasons, and they should not all be pushed through the same experimental sequence without adaptation. One of the main strengths of a microfluidic framework is precisely that it allows assay order to become compound-aware. Chemistry can be challenged before biology is overinterpreted; transport can be tested before neuronal rescue is assumed; timing can be made explicit before efficacy is generalized; and mechanistic profiling can be used to test coherence rather than to decorate a weak signal with additional endpoints. In this sense, the central contribution of microfluidics is not simply technological. It is interpretive: it improves how early-stage neuroprotective evidence is judged.

At the same time, the field should resist the temptation to claim more than the data currently support. Microfluidic neuroprotection has matured enough to justify a practical triage logic, but not enough to claim a universally validated end-to-end discovery pipeline. Direct CNS benchmarking remains uneven, exposure verification is still inconsistent, and reporting quality varies substantially across studies. These limitations do not diminish the value of the field; they define where the next gains must come from. More rigorous material-aware dosing, stronger transport measurement, clearer benchmark compounds, fit-for-purpose quality criteria, and better alignment between model complexity and the decision being asked are likely to improve the usefulness of these platforms more than simple escalation toward ever more elaborate systems. In particular, the current literature does not yet provide prospective evidence that a complete microfluidic neuroprotection pipeline reduces false-positive rates, enriches clinically viable candidates, or improves clinical concordance compared with a well-controlled conventional workflow; this remains a priority for future benchmarking studies.

The most persuasive future direction is therefore not maximal complexity, but selective integration. Human iPSC-derived BBB/NVU models, sensor-integrated chips, multicellular inflammatory assemblies and more mature organoid-linked systems will be most valuable when they are deployed at the point in the workflow where they remove a real interpretive uncertainty. Used in this way, microfluidics can help impose discipline on a field that has long struggled with false confidence, assay-dependent optimism and costly late-stage failure. The central conclusion is therefore not that microfluidics has already solved the translational problem in neuroprotection, but that it can make specific sources of uncertainty more visible. This Review deliberately separates what microfluidic systems can already measure more explicitly than static plate assays, where they can plausibly inform within-program prioritization, and what remains unproven until prospective benchmarking demonstrates improved downstream enrichment or stronger clinical concordance.

## Figures and Tables

**Table 2:** Microfluidic modalities for neuroprotection workflows and representative use cases.

Module	What It Physically Controls	Best-Supported Neuroprotection Use	Evidence Status in Compound-Centered Workflows
Gradient generators/laminar networks	Concentration history, wash-in/wash-out timing, reperfusion waveform	Dose-response mapping, penumbra-like patterns, controlled injury transitions	Established [58,59,60,61,71]
Compartmentalized neuron chips	Spatial separation of soma/axon and cell-cell interfaces	Axon-specific injury/repair, mitochondrial transport, neuron-glia crosstalk	Established [58,59,60,61,62]
Hydrodynamic traps/microwells	Standardized position and residence time for single objects, spheroids or organoids	Repeated imaging and exposure of scarce 3D samples under flow	Emerging to moderate [72,73,74]
DLD and inertial sorting	Label-free preprocessing and quality control of heterogeneous inputs	Cell-suspension cleanup, organoid-fragment QC, EV pre-fractionation	Emerging [75,76,77]
Electrokinetics (EOF/DEP)	Selective routing, pumping or preconcentration with electric fields	Sample preparation, local manipulation, electrode-compatible workflows	Emerging; much evidence remains adjacent rather than CNS-specific [77,78,79,80,81,82]
Acoustofluidics (SAW/BAW)	Gentle contactless separation and concentration	EV purification and diagnostic sample preparation	Emerging; stronger for biomarker prep than for direct compound ranking [75,83,84]
Magnetofluidics	Immunocapture and washing in complex fluids	Targeted biomarker pull-down and automated rare-sample handling	Emerging; strongest for workflow support [79,85]
Droplet microfluidics	Dense compartmentalization, rapid combinatorial dosing and sorting	Ultrahigh-throughput prescreens and narrow dose-window mapping	Established for prescreening; not a standalone predictor of neuroprotective efficacy [51,52,53,54,55,56]

**Table 3:** Mitochondria-anchored readouts for microfluidic neuroprotection assays.

Readout	Implementation	Best Decision Use	Common Confounders/Limits
Δψm	Fluorescent dyes; ratiometric probes; microelectrode methods	Early injury detection and recovery timing under OGD/reperfusion	Phototoxicity; dye loading variability; need calibration. Reference controls and calibration should accompany interpretation [24,60,97].
ROS/redox	Genetically encoded sensors (HyPer/roGFP); chemical dyes	Testing whether antioxidant claims survive time-resolved measurement	Probe specificity; redox-cycling compounds; light sensitivity. Orthogonal redox checks are especially important for redox-active compounds [27,60].
ATP/energy	Luminescence endpoints; microfluidic sampling; integrated sensors	Energetic rescue vs. mere structural survival	Endpoint bias; careful normalization to cell number required. Normalize to cell number and pair with viability/structural endpoints [60,73].
Ca^2+^ dynamics	Ca^2+^ indicators; stimulation under flow	Excitotoxicity and mitochondria-ER coupling under controlled dosing	Indicator buffering; perfusion artifacts; complex interpretation. Interpret alongside excitotoxicity and mitochondrial context [24,60].
Morphology and dynamics	Live imaging + automated analysis (e.g., MitoQuant)	Fragmentation, fusion/fission balance and delayed remodeling	Segmentation errors; standardized imaging conditions needed. Use standardized imaging and segmentation settings [32,60].
Mitophagy flux	Reporter constructs (e.g., mito-Keima); autophagy markers	Quality-control competence during delayed injury and recovery	Reporter burden; requires time-resolved design. Requires time-resolved flux design rather than endpoint staining alone [31,32].
Axonal mitochondrial transport	Compartmentalized chips + time-lapse tracking	Dying-back vulnerability and synaptic maintenance	Requires stable axon outgrowth; imaging/data burden is high. Best supported in compartmentalized neuronal formats [58,59,60,61,62].

**Table 4:** Staged compound-triage framework with illustrative within-program pre-specification examples only (not field benchmarks, decision cutoffs, or consensus thresholds).

Stage	Primary Decision Question	Representative Platform/Readouts	Illustrative Program-Level Pre-Specification Example (Examples Only; Adapt Locally)
0	Is the chemistry assay-ready?	Library design + counterscreens: solubility, stability, orthogonal interference checks [27,38,39,40,51,52,53,54,55,56,57]	Program-defined assay ability window, e.g., parent compound largely preserved over the assay period, vehicle kept within a low tolerated range, and obvious interference liabilities flagged before biological testing.
1	Is there a reproducible concentration-response worth escalating?	Droplet prescreen: aggregation kinetics or ROS/Δψm proxy; dense dose-response [51,52,53,54,55,56]	Program-defined concentration-response quality rule, e.g., several informative concentrations away from ceiling/floor effects and replicate variability bounded in advance.
2	Does the compound survive transport and barrier safety constraints?	BBB/NVU filter: TEER, Papp, mass balance/recovery, inflammatory markers [43,49,50,63,64,97]	Program-defined exposure-and-safety window, e.g., measurable post-chip recovery with no material barrier deterioration beyond a locally justified tolerance.
3	Does efficacy persist under clinically relevant injury timing?	Neuronal microfluidic OGD/I/R: Δψm recovery, ROS, viability, neurite/axon integrity, transport [33,34,35,58,59,60,61,62]	Program-defined delayed-dosing criterion, e.g., benefit retained at reperfusion or a justified post-insult delay and reproduced across independent experimental batches.
4	Is there a coherent development hypothesis?	Mechanistic triangulation: mitochondria + barrier + function [41,42,49,63,64,65]	Program-defined coherence rule, e.g., at least two orthogonal readouts move compatibly without introducing incompatible barrier, toxicity or exposure liabilities.

**Table 5:** Retrospective decision examples and benchmark uses of selected microfluidic stages in neuroprotective compound triage. The examples indicate where microfluidic formats may change interpretation, prioritization or follow-up testing by exposing specific uncertainties in exposure, timing, barrier function, inflammation or compartment-specific mechanisms. They should be interpreted as evidence-anchored decision examples, not as prospectively validated proof of clinical predictivity.

Case/Use Scenario	Conventional-Workflow Uncertainty	How a Microfluidic Stage Can Change Interpretation	Example Control or Validation
NXY-059/edaravone -type acute redox rescue	A pretreatment or poorly timed static assay may overstate rescue when clinical treatment would occur during reperfusion or after delay.	A neuronal OGD/reperfusion chip plus BBB/NVU transport stage can test delayed dosing and brain-facing exposure before escalation.	Retrospective benchmark logic; not a demonstrated microfluidic reranking of clinical outcome [33,34,35,36,37,43,49,50].
Resveratrol/curcumin and related polyphenols	Optical artifacts, hydrophobic adsorption, formulation dependence, and hormesis can create false confidence in plate assays.	Droplet dose grids, counter screens, and material-recovery checks can derank chemically fragile signals before BBB/NVU or neuronal testing.	Established assay-liability rationale; compound-specific CNS microfluidic validation remains moderate [27,38,39,40,51,52,53,54,55,56,57].
Barrier- or neuroimmune -modulating agents	Neuron-only cultures may underrate compounds whose main effect is endothelial, pericyte, astrocytic, microglial, or leukocyte-linked.	Tri-culture BBB/NVU chips can reprioritize candidates when TEER/Papp, cytokines, or immune-cell interactions improve despite weak neuron-only potency.	Emerging direct support; requires benchmarking and transport verification [41,42,49,63,64,65].
Anti-aggregation/proteostasis modulators	Cell-free aggregation activity may not persist in neurons where trafficking, uptake, clearance, and propagation dominate.	Droplet aggregation assays can compress chemical uncertainty, while compartmentalized neuronal chips test propagation and living-cell context.	Mechanistic support, but not an end-to-end neuroprotection predictor [3,51,52,53,54,55,56,66,67].
Elamipretide/mitochondria-directed rescue	Bulk viability may appear improved even when Δψm recovery, ATP-linked function, axonal mitochondrial transport or delayed structural recovery remain incoherent.	A timed neuronal OGD/reperfusion chip can distinguish pretreatment-only mitochondrial stabilization from delayed, multiparametric rescue under the same injury geometry.	Mechanistic support; still requires prospective workflow benchmarking [24,32,48,58,59,60,61,62].
BBB-chip ischemia -reperfusion benchmark (Wu et al.)	Static OGD or Transwell-type assays may not synchronize BBB breakdown, apoptosis and mitochondrial dysfunction under a controlled reperfusion transition.	A BBB chip can impose ischemia/reperfusion-like switching and monitor barrier injury, cell death and mitochondrial dysfunction in one timed device, making the timing and multicellular injury logic explicit.	Direct platform benchmark for stroke-like I/R; useful for decision timing, not yet a compound-series reranking study [35].
Human Brain-Chip neuroinflammation benchmark (Pediaditakis et al.)	Neuron-only or Transwell assays may miss whether a candidate acts through endothelial, glial or inflammatory crosstalk rather than direct neuronal rescue.	A multicellular human Brain-Chip links TNF-alpha-induced barrier permeability, glial activation, cytokine release and transcriptomic similarity to adult cortex, supporting neuroimmune/barrier-oriented triage.	Direct human MPS benchmark; supports context -dependent interpretation but still requires compound-specific validation [42].
Perfusable 3D human NVU drug-delivery/immune -extravasation model (Qiu et al.)	Drug delivery, endothelial vessel perfusion and immune-cell entry are often tested separately, making it difficult to judge exposure and vascular safety together.	A perfused 3D NVU chip can couple brain-facing delivery with immune extravasation and neural-cell context, which is useful before advancing peptides, antibodies or nanocarriers.	Direct delivery/extravasation platform; decision value strongest for exposure and safety filters, not final efficacy prediction [63].
CNS-active compound perfusion/calcium-imaging benchmark (MacKerron et al.)	Bulk neuronal assays can show that a compound changes activity, but not whether direct and synaptically propagated responses are separable under repeatable perfusion.	Compartmentalized perfusion with calcium imaging can generate concentration-response and antagonist-control data while monitoring downstream synaptic network activity in an isolated chamber.	Direct pharmacological benchmark for CNS-active compounds; added as adjacent neuropharmacology evidence rather than a neuroprotection outcome predictor [100].

**Table 6:** Minimal reporting and quality-control checklist with illustrative within-program pre-specification examples only (adapt locally; not field benchmarks or consensus thresholds).

Domain	Minimum Item to Report	Illustrative Program-Level Pre-Specification Example (Adapt Locally)	Example Control or Validation
Device and materials	Substrate, coating, channel dimensions, membrane/hydrogel properties, flow rate and estimated shear	Program-defined recovery/mass-balance window appropriate to the material stack and analyte.	Tracer recovery, mass balance, surface-adsorption check [50,57,101]
Biology and maturation	Cell source, passage or differentiation batch, co-culture composition, maturation state, baseline phenotype	Program-defined baseline acceptance band for barrier or neuronal maturation metrics, with batch variability reported.	Baseline TEER/Papp, marker panel, spontaneous activity or morphology QC [43,49,64,101]
Injury paradigm	OGD composition, oxygen target, duration, reperfusion schedule, transition times	Program-defined injury-delivery verification window showing that the declared O_2_/glucose transition was actually achieved.	Dissolved O_2_ or sensor verification; glucose confirmation; time-stamped protocol logs [35,73]
Compound handling	Stock solvent, final solvent %, solubility, stability, free concentration when possible	Program-defined exposure verification rule, e.g., solvent cap and at least one direct check of stability, recovery or free concentration.	Inlet-outlet quantification; adsorption test; solvent-only control [50,57,101]
Readouts and decision rules	Core stage-matched readouts plus explicit go/no-go thresholds	Program-defined analysis rule with a predeclared primary endpoint, orthogonal confirmatory readout and missing-data/exclusion policy.	TEER/Papp on BBB chips; Δψm/ROS/ATP and axon integrity on neuronal chips [43,49,50,97]
Quality control and statistics	Bubble logs, exclusion criteria, randomization, interleaved controls, batch structure and replication	Program-defined replication and QC plan with independent batches, failed-run logging and transparent exclusions.	Benchmark compounds, failed-run log, blinded analysis or pre-defined analysis plan [49,64,101,102]

## Data Availability

No new data were created or analyzed in this study.

## Abbreviations

The following abbreviations are used in this manuscript:

ADP	adenosine diphosphate
ATP	adenosine triphosphate
Aβ	amyloid beta
BBB	blood-brain barrier
CNS	central nervous system
DEP	dielectrophoresis
DLD	deterministic lateral displacement
EOF	electroosmotic flow
EP	electrophoresis/electrophoretic manipulation
ER	endoplasmic reticulum
EV	extracellular vesicle
FAD	flavin adenine dinucleotide
I/R	ischemia-reperfusion
IDT	interdigital transducer
iPSC	induced pluripotent stem cell
MEA	microelectrode array
MPS	microphysiological system(s)
mPTP	mitochondrial permeability transition pore
NADH	nicotinamide adenine dinucleotide hydride
NF-κB	nuclear factor kappa-light-chain-enhancer of activated B cells
NRF2	nuclear factor erythroid 2-related factor 2
NVU	neurovascular unit
OCR	oxygen consumption rate
OECT	organic electrochemical transistor
OGD	oxygen-glucose deprivation
Papp	apparent permeability
PDMS	polydimethylsiloxane
PRISMA	Preferred Reporting Items for Systematic Reviews and Meta-Analyses
QC	quality control
roGFP	reduction-oxidation sensitive green fluorescent protein
ROS	reactive oxygen species
SAR	structure-activity relationship
SAW/BAW	surface/bulk acoustic waves
TEER	transendothelial electrical resistance
TNF-α	tumor necrosis factor alpha
α-syn	alpha-synuclein
Δψm	mitochondrial membrane potential
